# Development of a One-Step Real-Time TaqMan Reverse Transcription Polymerase Chain Reaction (RT-PCR) Assay for the Detection of the Novel Variant Infectious Bursal Disease Virus (nVarIBDV) Circulating in China

**DOI:** 10.3390/v15071453

**Published:** 2023-06-27

**Authors:** Chenyan Wang, Bo Hou, Guoqing Shao, Chunhe Wan

**Affiliations:** 1 Institute of Animal Husbandry and Veterinary Medicine, Fujian Academy of Agricultural Sciences, Fujian Animal Disease Control Technology Development Center, Fuzhou 350013, China; 15396022617@163.com (C.W.);; 2 Institute of Veterinary Medicine, Jiangsu Academy of Agricultural Sciences, National Research Center for Engineering and Technology of Veterinary Bio-Products, Nanjing 210014, China

**Keywords:** infectious bursal disease virus, one-step RT-PCR, real-time TaqMan, nVarIBDV

## Abstract

The novel variant IBDV (nVarIBDV, especially genotype A2dB1) mainly affects broilers in China. It causes an infection characterized by the atrophy of the bursa, a decrease in the level of lymphocytes, proliferation of fibrous tissue around the follicle, and severe atrophy of the follicle in the bursa. Poultry vaccinated with live IBDV vaccines do not have the challenge present with bursa atrophy, which is misdiagnosed for nVarIBDV because of the lack of other gross clinical symptoms. The present study sought to explore the potential and reliability of the real-time TaqMan analysis method for the detection and discrimination of the nVarIBDV genotype from that of the non-nVarIBDV, especially in live vaccine strains. This method will help monitor vaccinated poultry to control and manage infection with the nVarIBDV IBDVs. The nucleotide polymorphism in the 5′-UTR region and the *vp*5/*vp*2 overlapping region of the segment A sequences of IBDV were used to establish a one-step real-time TaqMan reverse transcription polymerase chain reaction (RT-PCR) method in this study. The results showed that the method accurately distinguished the nVarIBDV and non-nVarIBDV strains (especially live vaccine strains), and there were no cross-reactions with the infectious bronchitis virus (IBV), Newcastle disease virus (NDV), avian influenza virus (AIV), infectious laryngotracheitis virus (ILTV), fowlpox virus (FPV), *Mycoplasma gallisepticum* (*M. gallisepticum*), *Mycoplasma synoviae* (*M. synoviae*), and IBDV-negative field samples. The method showed a linear dynamic range between 10^2^ and 10^7^ DNA copies/reaction, with an average R^2^ of 0.99 and an efficiency of 93% for nVarIBDV and an average R^2^ of 1.00 and an efficiency of 94% for non-nVarIBDV. The method was also used for the detection of 84 clinical bursae of chickens vaccinated with the live vaccine. The results showed that this method accurately distinguished the nVarIBDV and non-nVarIBDV strains (vaccine strains), compared with a strategy based on the sequence analysis of HVRs at the *vp*2 gene or the reverse transcription PCR (RT-PCR) for the *vp*5 gene. These findings showed that this one-step real-time TaqMan RT-PCR method provides a rapid, sensitive, specific, and simple approach for detection of infections caused by nVarIBDV and is a useful clinical diagnostic tool for identifying and distinguishing nVarIBDV from non-nVarIBDV, especially live vaccine strains.

## 1. Introduction

Infectious bursal disease (IBD) is a common viral disease that affects poultry worldwide. Infectious bursal disease virus (IBDV) has a bi-segmented RNA genome (segments A and B) located in a nonenveloped icosahedral capsid. The virus belongs to the *Avibirnavirus* genus in the Birnaviridae family and was initially reported in Gumboro, USA in 1957 [1]. Studies on neuralization and cross-protection experiments report two serotypes of IBDV. Serotype 1 strains cause infections in chicken. These strains are classified as attenuated strains, classical strains (also known as standard strains), variant strains of USA, very virulent (vv) strains [2,3], and novel variant IBDV (nVarIBDV) [4,5,6], according to their pathogenicity. Serotype 2 strains are nonpathogenic to poultry [6]. Segment A consists of two overlapping open reading frames (ORF). The ORF1 encodes viral non-structural proteins (VP5), while ORF2 encodes a polyprotein (VP2-VP4-VP3) that can be cleaved through autoproteolysis to produce VP2, VP3, and VP4 [7]. VP2 forms the outer surface of the virion and serves as a major immune activation protein that can induce host neutralization antibodies. The *vp*2 gene has a hypervariable region (HVR, nt 616–1050), which is responsible for the viral antigenic variation and virulence [8,9]. Genomic segment B (2.9 kbp) encodes the RNA-dependent RNA polymerase VP1 [10].

The classic and vvIBDV strains are associated with different levels of mortality, with vvIBDV strains characterized by higher morbidity and mortality than classic strains. vvIBDV strains cause a mortality rate of 40–100% in specific pathogen-free (SPF) chickens, up to 60% in layers, and up to 30% in broilers [11,12,13]. Additionally, the vvIBDV was found in the natural infection of turkey poults for the first time in Egypt [14]. Major symptoms of the disease include muscular hemorrhage, inflammatory exudation, hemorrhage, and yellow staining of the bursae of poultry infected with classic and vvIBDV strains [5,13,15]. The nVarIBDV strains (such as FJ2019-01 and SHG19) are novel pathogenic viruses and are significantly different from the American IBDV variants. These viruses are characterized by the atrophy of the bursa, a decrease in the levels of lymphocytes, macrophage infiltration in the follicle, proliferation of fibrous tissue around the follicle, and severe atrophy of the follicle in SPF chickens [4,5]. Notably, no gross clinical symptoms or mortality were observed in the chicken infected with SHG19 or FJ2019-01 IBDV variants [4,5]. Currently used commercial IBDV vaccines are not effective against infection by these nVarIBDVs [5,16]. The nVarIBDVs cause subclinical infections that increase the susceptibility to infection by other pathogens and induce a poor immune response to vaccines [4].

Vaccination is the most effective control of IBD. However, studies report that poultry vaccinated with immune complex vaccines or live vaccines before infection with the virus presented with atrophy of the bursae, compared with that of non-vaccinated birds [17], and had an increased susceptibility to other pathogens [18]. The nVarIBDVs are not effective for differentiation between infected and vaccinated animals (DIVA). Therefore, it is imperative to explore a strategy for the differentiation of infected animals from vaccinated animals for IBDV for effective monitoring of vaccinated flocks to control infections with the nVarIBDVs. The aim of this study was to evaluate the potential and reliability of one-step real-time TaqMan analysis of the 5′-UTR region and the *vp*5/*vp*2 overlapping region of the segment A sequences of IBDV to detect and discriminate the genotype of the nVarIBDV from that of the non-nVarIBDV using allelic discrimination probes, especially live vaccine strains. The findings from the study will provide a basis for improving the current diagnostic capability, and the approach is a rapid, sensitive, and specific method for the effective screening of a large number of samples and for distinguishing the vaccine strains that cause bursae atrophy.

## 2. Materials and Methods

### 2.1. Virus Strains

The IBDV FJ2019-01 strain (GenBank: MZ736578 or MZ044944) was stored in our laboratory [5]. The IBDV BC6/85 strain (GenBank: ON286951) was maintained in our laboratory and used to assess the effectiveness of licensed IBDV vaccines currently in use in China [5]. The licensed IBDV live vaccine strains, B87, D78, W2512, M.B., K85, NF8, and CF, and BC6/85, YM (vvIBDV), WH (vvIBDV), FJ2019-01, FJ2019-02 (GenBank: MZ044945), FJ2019-03 (GenBank: MZ044946), FJ2019-04 (GenBank: MZ044947), FJ2019-05 (GenBank: MZ044948), and FJ2021(GenBank: MZ593902) from China were used to evaluate the specificity of the assay. RNA or DNA were extracted from the Newcastle disease virus (NDV), avian influenza virus (AIV), infectious laryngotracheitis virus (ILTV), fowlpox virus (FPV), *Mycoplasma gallisepticum* (*M. gallisepticum*), *Mycoplasma synoviae* (*M. synoviae*), and infectious bronchitis virus (IBV) to carry out specificity tests.

### 2.2. Primer and Probe Designs

**Figure 1:**
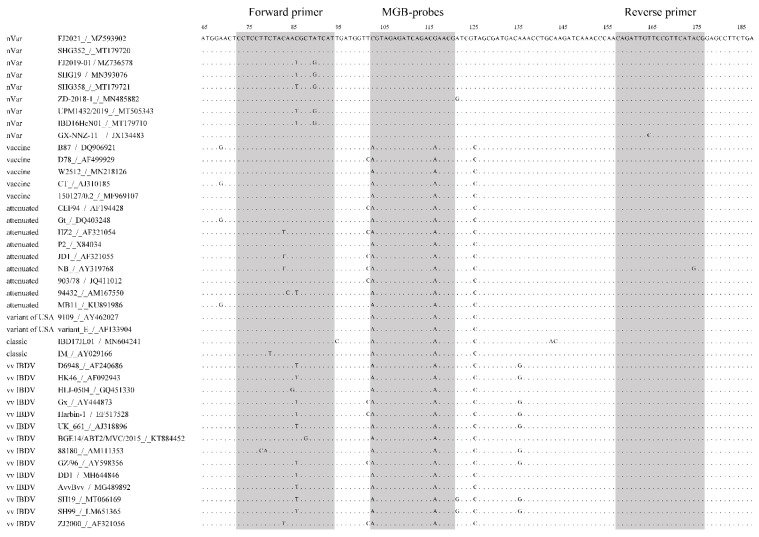
Alignment of the TaqMan-minor groove-binding (MGB) real-time reverse transcription polymerase chain reaction (RT-PCR)-amplified region. Various representative nVarIBDV and non-nVarIBDV isolates were included in the alignment. Forward and reverse primers and the probe target site are indicated using shadow position of the reference strain FJ2021 (MZ593902) in the alignment.

Complete and partial sequences of IBDV genomes (approximately 80 sequences) were retrieved from the GenBank database. Multiple sequence alignments were carried out using MEGA7 software. A nucleotide polymorphism that distinguished the nVarIBDV genogroup from other IBDV strains (including the classic, vvIBDV, variants of USA, and live vaccine strains) was explored. Primer sets and TaqMan minor groove-binding (MGB) probes were designed, based on the 5′-UTR region and the *vp*5/*vp*2 overlapping region of the segment A sequences, according to highly conserved regions observed in the multiple sequence alignment (Figure 1), for subsequent use in real-time qPCR. The primer sequences F: 5′-CCT CCT TCT AYA RYG CTR TCA T-3′ and R: 5′-CGT ATG AAC GGA ACA ATC TG-3′ were used to target the SNP-containing region and to amplify a 105 bp sequence (Figure 1). The A/G change maximized differences in Tm between allele-specific probes. Therefore, the probe sequences used in the study were 5′-TAG AGA TCA GAC GAA CG-3′, corresponding to nVarIBDV labeled with the VIC and MGB quencher groups in the 5′ and 3′ termini (named PnV), and 5′-AGT AGA GAT CAG ACA AA-3′, corresponding to non-nVarIBDV labeled with the FAM and MGB quencher groups in the 5′ and 3′ termini (named PnnV), respectively. The primers and probes were synthesized by Sangon Biotech Co., Ltd. (Shanghai, China). 

### 2.3. Viral RNA Extraction and Reverse Transcription

Viral RNA extraction from samples was performed using the QIAamp Viral RNA Mini Kit (Qiagen, Hilden, Germany), according to the manufacturer’s instructions. RNA from vaccines was extracted using PBS resuspension of lyophilized vaccine powder. Reverse transcription (RT) was performed with the RevertAid First Strand cDNA Synthesis Kit (Thermo Fisher Scientific, Waltham, MA, USA). 

### 2.4. Construction of a Positive Plasmid Standard

Partial segment A of the FJ2019-01 strain and the BC6/85 strain was amplified using the RevertAid RT kit (Thermo Fisher Scientific) with specific primers (10 μM; AU [5′-GGA TAC GAT CGG TCT GAC CCC GGG GGA GTC-3′] and A1542L [5′-GTA GTC TAC ACC TTC CCC AAT TGC AT-3′]) [19]. The purified PCR product was cloned into the pCRTMⅡ-Blunt-TOPO^®^ vector using the Zero Blunt^®^ TOPO^®^ PCR cloning kit (Invitrogen, Carlsbad, CA, USA), and the ligation product was used for the transformation of *E. coli*, DH5α (Tiangen, Beijing, China). The plasmid DNA was extracted using the Qiagen plasmid Mini kit (Qiagen, Hilden, Germany), and the concentrations of plasmids were determined using the NanoDrop 2000 spectrophotometer (Thermo Fisher Scientific). The target copy number of positive plasmids was calculated using the formula: copies/μL = (concentration in ng × 6.023 × 10^23^)/(genome length × 1 × 10^9^ × 660 dalton/bp). The pCR-BC6/85-A and pCR-FJ2019-01-A were constructed and used as positive controls for the detection of IBDV and for performing detection limit assay.

### 2.5. Establishment of the One-Step Real-Time TaqMan RT-PCR Method

The one-step real-time TaqMan RT-PCR method was performed on a LightCycler96 (Roche, Basel, Switzerland) to distinguish the nVarIBDV genogroup from the non-nVarIBDV strains (including the classic, vvIBDV, variants of USA, and live vaccine strains). The 20 μL reaction mix contained 5 μL Fast one-step Master Mix (4×) (Thermo Fisher Scientific), 0.50 μL forward or reverse primer (10 μM), 0.25 μL PnV probe (10 μM) or the PnnV probe (10 μM), 5 μL template RNA or plasmid DNA, and 8.5 μL deionized distilled water. The reaction conditions were as follows: 50 °C for 10 min; 95 °C for 20 s; 40 cycles of 95 °C for 3 s; and 60 °C for 30 s. Fluorescence intensities for specific reporter fluorophores were determined at the 60 °C step of each cycle and at the end of the run. All reactions and amplifications were performed and analyzed using the LightCycler^®^ 96 SW 1.1 software. 

### 2.6. Determination of Specificity of Primers and Probe Sets

The specificities of the primer pairs and probes were performed using NCBI BLAST https://www.ncbi.nlm.nih.gov/tools/primer-blast/ (accessed on 18 September 2021) (Bethesda, MD, USA). A BLAST search was performed to predict the in silico primer and probe sequence specificities and to evaluate the occurrence of non-specific homology between the sequences and the IBDV genome or the chicken genome. Previously confirmed IBDV-negative samples were also analyzed. The specificity of the established method was verified using RNA samples isolated from B87, D78, W2512, M.B, K85, NF8, CF, BC6/85, FJ2019-01, FJ2019-02, FJ2019-03, FJ2019-04, FJ2019-05, FJ2021, WH, and YS and RNA or DNA samples from NDV, IBV, AIV, ILTV, FPV, *M. gallisepticum,* and *M. synoviae*.

### 2.7. Assay Detection Limit

Serial dilutions (2 × 10^6^ copies/μL to 2 × 10^1^ copies/μL) of two positive plasmids prepared using deionized water were added in the respective reaction mix to evaluate the sensitivities of primers and probes. A standard curve was generated by plotting the Cq values vs. log10 of 10-fold serial dilutions (10^7^ to 10^2^) of plasmid DNA. Analyses for relative and absolute sensitivities were conducted in triplicate. The standard curve and assay efficiency were established using the LightCycler^®^ 96 SW 1.1 software. 

Interference resulting from differences in the proportion of the mixed templates was evaluated by mixing two positive plasmids in various ratios (1:10^7^, 1:10^6^ 1:10^5^, 1:10^4^, 1:10^3^, 1:10^2^, 1:0, 10^7^:1, 10^6^:1, 10^5^:1, 10^4^:1, 10^3^:1, 10^2^:1, and 0:1; 1 = 10^5^ copies/reaction). Comparisons of different mixed templates and single templates were performed using the obtained Cq values.

### 2.8. Clinical Sample Testing

A total of 84 clinical bursal samples were collected from 20- to 40-day-old chickens vaccinated with the live vaccine of W2512, B87, D78, M.B., K85, and CF strains obtained from an infection in 6 chicken flocks reported in 2021 in China. The samples were used to verify the accuracy of the established one-step real-time TaqMan RT-PCR assay. Virus RNA was extracted from bursal homogenates and analyzed using the one-step real-time TaqMan RT-PCR assay. The reverse transcription PCR assays were carried out with an IBDV fluorescent reverse transcription PCR (RT-PCR) detection kit (BIOTECHSY, Beijing, China) to detect the IBDV of the *vp*5 gene, which is present in all IBDVs, regardless of the genotype, to verify the results. In addition, PCR of HVRs at the *vp*2 gene was performed using the VP2-F (5′-CCT CAG CTT ACC CAC ATC-3′) and VP2-R (5′-CCT TCC CCA ATT GCA TGG-3′) primers, as described previously [4]. PCR products were sequenced and analyzed using previously reported methods [4,20]. 

## 3. Results

### 3.1. Feasibility of the One-Step Real-Time TaqMan RT-PCR Method

A BLAST analysis was performed for the segment A sequences of several IBDV strains to identify nucleotide polymorphisms that could be used to distinguish between nVarIBDV and non-nVarIBDV strains. Two single nucleotide polymorphisms (SNPs) were detected in this study that could be used to distinguish nCv and non-strains, based on the *vp*5 region. The C103A and G117A SNPs were selected for their high consistency to differentiate the genogroup from non-strains (including cIBDV, vvIBDV, variants of USA, and live vaccine strains) (Figure 1). This was important because the A/G change maximized Tm differences between allele-specific probes. Two TaqMan-MGB probes were designed to discriminate and separately quantify the nVarIBDV and non-nVarIBDV genotypes. The PnV probe was labeled with the VIC fluorescent dye and was specific for nVarIBDV strains. The PnnV probe was labeled with the 6-FAM fluorescent dye and was specific for non-nVarIBDV strains. 

**Figure 2:**
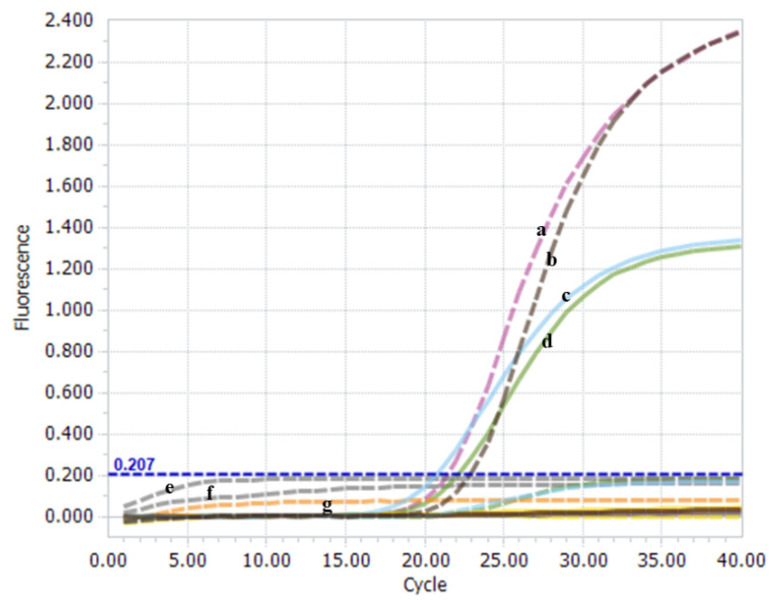
The feasibility of the one-step real-time TaqMan RT-PCR method. IBDV FJ2019-01 strain (a), positive plasmid pCR-FJ2019-01-A (b), BC6/85 strain (c), and positive plasmid pCR-BC6/85-A (d) specifically exhibited the amplified VIC signal (dotted line) and the FAM signal (full line). The IBDV-negative field sample (e), AVE buffer (f), and H_2_O (g) did not exhibit amplified signals during the assay.

A one-step real-time TaqMan RT-PCR platform was established to simultaneously discriminate and quantify nVarIBDV and non-nVarIBDV genotypes. The results showed that only the positive plasmid of nVarIBDV was recognized on the VIC channel, and the positive plasmid of non-nVarIBDV was recognized on the FAM channel (Figure 2). Notably, no significant cross-reaction was observed between positive plasmids of nVarIBDV and non-nVarIBDV using the non-specific TaqMan-MGB probe (Figure 2). The amplification from nVarIBDV (FJ2019-01) and non-nVarIBDV (BC6/85) was detected only with matching primer and probe combinations, indicating typical “S” amplification curves. The Cq values were all less than 35. No specific amplification was detected from mismatched combinations of probes. Negative controls did not exhibit amplification curves (Figure 2). These findings indicate that the established one-step real-time TaqMan RT-PCR method is highly feasible and suitable for the simultaneous identification of the viral RNA of nVarIBDV and non-nVarIBDV.

### 3.2. Specificity of the One-Step Real-Time TaqMan RT-PCR Method

**Figure 3:**
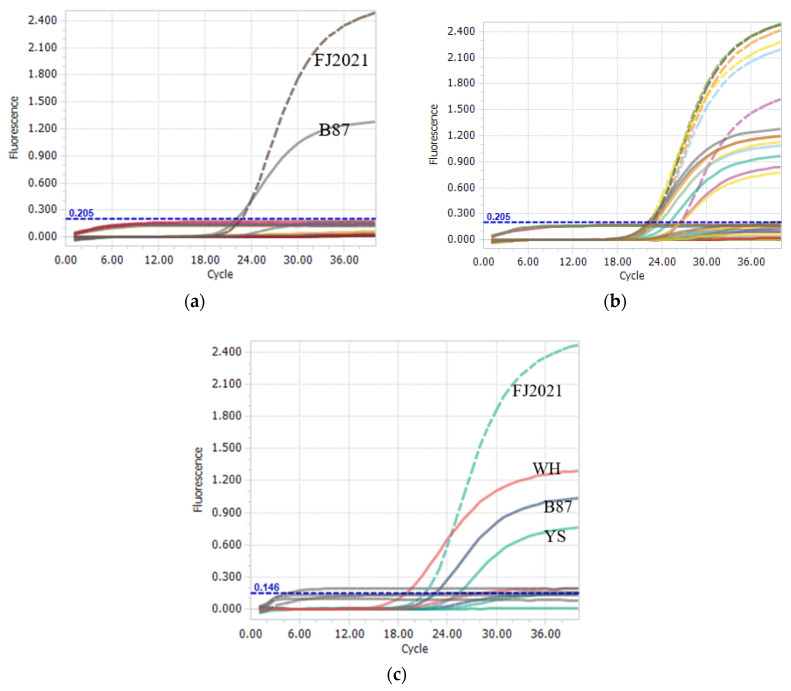
Specificity of the one-step real-time TaqMan RT-PCR assay. (**a**) FJ2021 and B87 strains were the positive controls for VIC and FAM signals, respectively; IBV, NDV, AIV, ILTV, FPV, *M. gallisepticum*, *M. synoviae,* and IBDV-negative field samples did not show amplification signals for VIC or FAM during the assay. (**b**) Six nVarIBDV strains, BC6/85, and seven vaccine strains were analyzed to specifically generate the amplified VIC signal and FAM signal, respectively. (**c**) Two vvIBDV strains (WH and YS) were analyzed to specifically generate the amplified FAM signal.

A nucleotide BLAST search of each primer and probe only showed homology with the genome regions of the expected IBDV genogroup strain. The IBDV-negative field samples and non-IBDV pathogenies (IBV, NDV, AIV, ILTV, FPV, *M. gallisepticum,* and *M. synoviae*) were not amplified, and the negative or blank controls in the assay were also not amplified (Figure 3a). Six nVarIBDV strains, two vvIBDV strains, and seven vaccine strains were analyzed to evaluate the assay performance and diagnostic effectiveness (Figure 3b,c). All strains were accurately diagnosed using this method, indicating 100% sensitivity and effectiveness of the method. This result indicates that the two probes were highly specific, feasible, and suitable for targeting the IBDV genogroup, and no cross-reactivity was observed between the nVarIBDV and non-nVarIBDV strains. 

### 3.3. Sensitivity of the One-Step Real-Time TaqMan RT-PCR Method

**Figure 4:**
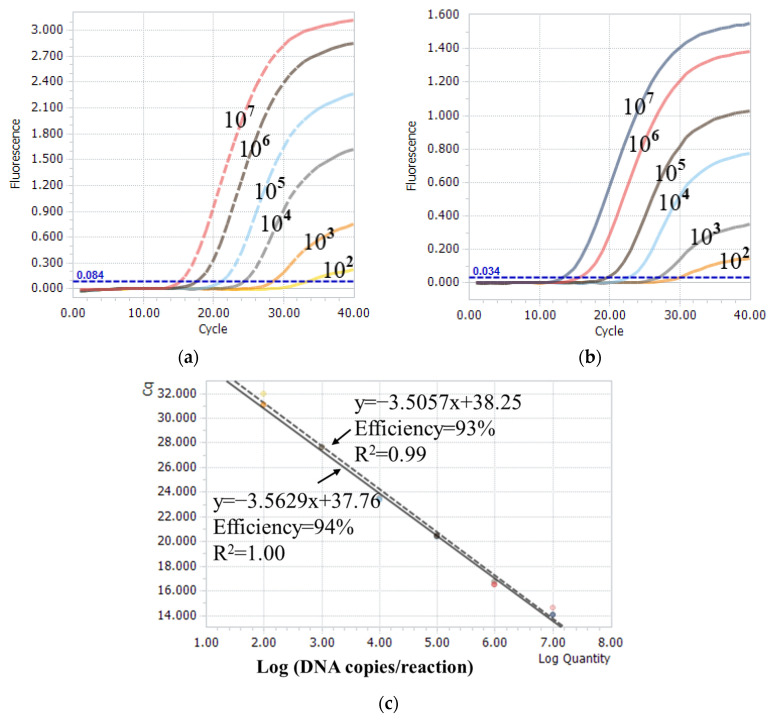
Sensitivity and standard curves of the one-step real-time TaqMan RT-PCR assay. (**a**,**b**) Amplification curves using 10-fold serially diluted template concentrations of pCR-FJ2019-01-A and pCR-BC6/85-A plasmids. (**c**) The linear dynamic range was established between 10^2^ and 10^7^ copies/reaction for nVarIBDV (dotted line) and non-nVarIBDV (full line) templates. The coefficient of determination (R^2^) and efficiency of each linear regression curve are indicated.

The one-step real-time TaqMan RT-PCR method was performed using a series of known concentrations of plasmid standards to explore its sensitivity. The assay using the pCR-FJ2019-01-A plasmid as a template showed a linear dynamic range between 10^2^ and 10^7^ DNA copies/reaction, with an average R^2^ of 0.99 and an efficiency of 93% (Figure 4a,c). The assay using the pCR-BC6/85-A plasmid as a template exhibited a linear dynamic range between 10^2^ and 10^7^ DNA copies/reaction, with an average R^2^ of 1.00 and an efficiency of 94% (Figure 4b,c). Additionally, the assay was performed using different ratios of the nVarIBDV template to non-nVarIBDV template. The Cq values obtained from the assay are presented in Table 1. Compared with single templates, low-copy (less 10^3^ copies/reaction) templates did not generate the amplified FAM signal or VIC signal when the mixed templates had another template with high copies. This finding implied a presence of competition for low copies of templates.

### 3.4. Analysis of the Clinical Samples Using the One-Step Real-Time TaqMan RT-PCR Method

A total of 84 clinical bursae samples were collected from six chicken flocks vaccinated with W2512, B87, D78, M.B., K85, and CF strains. The results showed that 84 samples were positive for the one-step real-time TaqMan RT-PCR method (84/84, 100%) (Table 2). This implies that the established one-step real-time TaqMan RT-PCR method can be used for nVarIBDV and non-nVarIBDV strain detection. A total of 19 samples out of the 84 clinical samples were positive for nVarIBDV (22.62%), 67 samples were positive for non-nVarIBDV (79.76%), and 2 samples were positive for both IBDV (2.38%) (Table 2). Then, RT-PCR was conducted for the vp5 gene to detect IBDV, and the results were positive for all 84 clinical bursae samples. Sequence analysis of HVRs at the vp2 gene was conducted to explore the genotyping capacity of the method. Some IBDV-positive samples (15 of 84) failed the sequence analysis of the HVRs process, due to a low number of copies of the templates. The results showed that IBDV-positive samples that were successfully sequenced were correctly detected by the corresponding specific TaqMan-MGB probe to the genotype (Table 2). This finding demonstrated that the established one-step real-time TaqMan RT-PCR method has a potential genotyping capacity and high effectiveness. The specificity and sensitivity of the one-step real-time TaqMan RT-PCR method were 100% and 100%, respectively, compared with the traditional sequence analysis of HVRs at the vp2 gene (Table 2). The genotype of IBDV is important in distinguishing between the vaccine strains with atrophied bursae and nVarIBDV and can be quickly explored using the one-step real-time TaqMan RT-PCR method, compared with traditional RT-PCR and sequence analysis.

## 4. Discussion

IBD induced by serotype 1 IBDV is a common infectious disease reported in the chicken industry globally and was also found in turkey poults for the first time in Egypt [14]. Serotype 1 strains are classified as avirulent strains, classical strains (also known as standard strains), variant strains of USA, and very virulent (vv) strains [2,3], according to their pathogenicity. The nVarIBDV strains belonging to the A2dB1 genotype were reported in 2019 in China and have rapidly spread to several provinces in China [4,5,6]. The new reassortment strains (genotype A2dB3) have also been observed in China, with a segment A from nVarIBDV strains and a segment B from HLJ0504-like strains (Genotype A3B3), which shows a similar pathogenicity to nVarIBDV in specific-pathogen-free (SPF) chickens [21]. Currently, various methods are available for the diagnosis of IBDV, such as virus isolation, RT-PCR, real-time RT-PCR, and ELISA. Real-time RT-PCR methods are highly sensitive and specific and mainly require “SYBR Green” or “TaqMan” probes to generate high-resolution melting curves or specific fluorescent signals. The real-time RT-PCR method is currently one of the most promising and effective methods used in control and epidemiological surveillance programs [22,23,24,25,26]. In addition, genetic relatedness is currently widely used to characterize IBDV strains [22,23,25,27,28]. Conventional reverse transcription polymerase chain reactions (RT-PCR) and diverse genotyping techniques, such as nucleotide sequencing and restriction fragment length polymorphism (RFLP) [20,22,29,30], have been replaced with the RT-PCR method using two distinct and specific probes or high-resolution melting curve analysis. This novel RT-PCR method can simultaneously and accurately discriminate differentiations between infectious bursal disease virus strains [22,25,26]; thus, it is more time-saving and economical, compared with conventional methods. Therefore, simpler and more rapid assays for detection and discrimination of all nVarIBDV from other IBDV genotype strains, especially vaccine strains, should be explored to monitor strain spread and improve the control of this disease. Differentiation of vaccines and wild strains is crucial for developing effective control strategies.

Monitoring of nVarIBDV is mainly conducted through sequencing of the full viral genome or sequencing partially conserved regions of *vp*1 and *vp*2 genes [4,5]. However, this approach is costly, requires technical expertise, and is characterized by long processing times, which delay control and treatment actions. In this study, an investigation of distinct SNPs at the *vp*5/*vp*2 overlapping region of the segment A sequences was conducted to evaluate them as potential targets to rapidly screen and distinguish nVarIBDV from non-nVarIBDV strains, especially live vaccine strains. Specificity of primers and two probes for the one-step real-time TaqMan RT-PCR method was evaluated experimentally, as well as through BLAST analysis. The results demonstrated that these primers and probes had no cross-reactions with virus DNA or RNA and the chicken genome. Moreover, the probes did not exhibit cross-reactions between nVarIBDV and non-nVarIBDV strains, indicating that the method had high specificity. The results using this novel assay showed that the set of primers and probes accurately distinguished the nVarIBDV and non-nVarIBDV strains, and there were no cross-reactions with IBV, NDV, AIV, ILTV, FPV, *M. gallisepticum*, *M. synoviae,* and IBDV-negative field samples. 

The real-time PCR method has a high detection limit and can be used for IBDV detection or for distinguishing vvIBDV from other non-vvIBDV genotype strains [22,25,26,31]. The method developed in the present study had a lower limit of detection for IBDV identification (10^2^ copies/reaction), compared with the traditional real-time PCR method. The findings showed that the method could detect target templates at a ratio of 10^5^:10^4^ copies/reaction of the two templates to accurately distinguish strains in the interference assay. The high Cq values of the target and no Cq (no amplification) to lower number of target copies in the mixed templates could be attributed to the interference of exponential amplification, due to a higher number of copies of target templates. However, the interference was not evaluated for mixed templates in the TaqMan-MGB real-time RT-PCR assay, which can accurately distinguish all vvIBDV strains from non-vvIBDV strains [22]. The results showed that the method simultaneously determined the identities of nVarIBDV and non-nVarIBDV strains (especially live vaccine strains) in bursa samples, even at a low target template concentration of 10^2^ copies/reaction.

Classic IBDV and vvIBDV infections cause hemorrhage, waxy yellow jelly in the bursa, and/or mortality [5,13,15]. Therefore, it is important to explore the differences in clinical symptoms between classic or vvIBDV strains and live vaccine strains in infected animals using different PCR methods to determine all strains of IBDVs. The nVarIBDV infection causes atrophy of the bursa, induces a decrease in the level of lymphocytes, promotes macrophage infiltration in the follicle, causes proliferation of fibrous tissue around the follicle, and leads to severe atrophy of the follicle in SPF chickens [4,5]. Notably, unchallenged chickens vaccinated with immune complex vaccines present with bursa atrophy with a bursa:body weight index (BBIX = [bursa:body weight ratios]/[bursa:body weight ratios in the negative group]) of 0.59–0.26, compared with those of unvaccinated birds aged 21 to 35 days [17]. Atrophy of the bursa and non-specific gross clinical symptoms minimized the differentiation of the nVarIBDV and live vaccine strains when reverse transcription PCRs (RT-PCR), nanoparticle-assisted PCR, SYBR green, and TaqMan-based real-time RT-PCRs (RT-qPCR) were used to detect all IBDVs [22,25,26,31,32]. The one-step real-time TaqMan RT-PCR method reported in this study effectively and simultaneously distinguished the nVarIBDV from commercially live IBDV vaccine strains and the clinical samples obtained from chickens vaccinated using the live vaccine. The results showed that some bursa samples obtained from chicken were effectively distinguished from the nVarIBDV and vaccine strains. These results imply that the one-step real-time TaqMan RT-PCR method is a highly sensitive, specific, and accurate method for simultaneous and accurate detection of the nVarIBDV in bursa samples obtained from chickens vaccinated with live vaccines when infection with the nVarIBDV is suspected. However, reassortment, gene mutations, and homologous recombination are the main mechanisms in the evolution of double-stranded RNA; the one-step real-time TaqMan RT-PCR method may have deficiencies when detecting the new IBDV variants.

## 5. Conclusions

This one-step real-time TaqMan RT-PCR method provides a rapid, sensitive, specific, and simple strategy for the detection of infections caused by nVarIBDV and for epidemiological investigation. Additionally, this method is a useful clinical diagnostic tool for identifying and distinguishing nVarIBDV from non-nVarIBDV, especially live vaccine strains.

## Figures and Tables

**Table 1:** Mean Cq values of different concentrations of the ratios of templates for nVarIBDV to non-nVarIBDV, as assessed using the one-step real-time TaqMan RT-PCR method.

Ratios of nVarIBDV to Non-nVarIBDV Templates (Copies/Reaction)	Cq FAM	Cq VIC
10^5^:10^7^	14.07	18.94
10^5^:10^6^	18.53	21.04
10^5^:10^5^	20.56	21.01
10^5^:10^4^	23.33	20.87
10^5^:10^3^	Negative	20.50
10^5^:10^2^	Negative	21.07
0:10^7^	14.06	Negative
0:10^6^	16.49	Negative
0:10^5^	20.44	Negative
0:10^4^	23.41	Negative
0:10^3^	27.60	Negative
0:10^2^	31.04	Negative
10^7^:10^5^	26.77	14.08
10^6^:10^5^	21.65	18.07
10^5^:10^5^	21.13	21.47
10^4^:10^5^	20.98	24.33
10^3^:10^5^	21.57	Negative
10^2^:10^5^	20.81	Negative
10^7^:0	Negative	14.58
10^6^:0	Negative	16.78
10^5^:0	Negative	20.32
10^4^:0	Negative	23.53
10^3^:0	Negative	27.66
10^2^:0	Negative	31.95

**Table 2:** Results of the clinical sample analyzed using the one-step real-time TaqMan RT-PCR method, the analysis of HVRs at the vp2 gene, or reverse transcription PCR (RT-PCR) for the vp5 gene.

Sample No.	One-Step Real-Time TaqMan RT-PCR		RT-PCR for vp5 Gene	HVRs of vp2 Gene
	FAM Cq	VIC Cq		
S1	negative	20.23	positive	nVarIBDV
S2	28.39	negative	positive	non-nVarIBDV
S3	29.95	negative	positive	N.D.
S4	32.25	negative	positive	N.D.
S5	27.83	negative	positive	non-nVarIBDV
S6	25.44	negative	positive	non-nVarIBDV
S7	29.95	negative	positive	N.D.
S8	24.01	negative	positive	non-nVarIBDV
S9	26.63	negative	positive	non-nVarIBDV
S10	27.22	negative	positive	non-nVarIBDV
S11	25.54	negative	positive	non-nVarIBDV
S12	28.67	negative	positive	N.D.
S13	negative	22.28	positive	nVarIBDV
S14	26.62	negative	positive	non-nVarIBDV
S15	29.11	negative	positive	N.D.
S16	27.55	negative	positive	non-nVarIBDV
S17	26.71	negative	positive	non-nVarIBDV
S18	28.95	negative	positive	N.D.
S19	30.13	negative	positive	N.D.
S20	25.46	negative	positive	non-nVarIBDV
S21	27.02	negative	positive	non-nVarIBDV
S22	25.18	negative	positive	non-nVarIBDV
S23	22.93	negative	positive	non-nVarIBDV
S24	27.36	negative	positive	non-nVarIBDV
S25	negative	23.39	positive	nVarIBDV
S26	26.49	negative	positive	non-nVarIBDV
S27	28.16	negative	positive	non-nVarIBDV
S28	25.77	negative	positive	non-nVarIBDV
S29	26.32	negative	positive	non-nVarIBDV
S30	29.15	negative	positive	N.D.
S31	27.03	29.80	positive	non-nVarIBDV
S32	21.43	negative	positive	non-nVarIBDV
S33	25.75	negative	positive	non-nVarIBDV
S34	23.13	negative	positive	non-nVarIBDV
S35	negative	22.59	positive	nVarIBDV
S36	29.10	negative	positive	N.D.
S37	26.70	negative	positive	non-nVarIBDV
S38	30.75	negative	positive	N.D.
S39	26.60	negative	positive	non-nVarIBDV
S40	29.02	negative	positive	N.D.
S41	23.34	negative	positive	non-nVarIBDV
S42	29.06	negative	positive	N.D.
S43	22.77	negative	positive	non-nVarIBDV
S44	26.68	negative	positive	non-nVarIBDV
S45	negative	26.02	positive	nVarIBDV
S46	26.33	negative	positive	non-nVarIBDV
S47	27.21	negative	positive	non-nVarIBDV
S48	25.83	negative	positive	non-nVarIBDV
S49	25.96	negative	positive	non-nVarIBDV
S50	negative	15.19	positive	nVarIBDV
S51	negative	14.33	positive	nVarIBDV
S52	22.24	negative	positive	non-nVarIBDV
S53	negative	11.37	positive	nVarIBDV
S54	negative	21.53	positive	nVarIBDV
S55	negative	18.45	positive	nVarIBDV
S56	27.71	negative	positive	non-nVarIBDV
S57	32.14	29.23	positive	N.D.
S58	negative	16.56	positive	nVarIBDV
S59	24.82	negative	positive	non-nVarIBDV
S60	negative	21.17	positive	nVarIBDV
S61	23.13	negative	positive	non-nVarIBDV
S62	25.93	negative	positive	non-nVarIBDV
S63	26.15	negative	positive	non-nVarIBDV
S64	28.01	negative	positive	non-nVarIBDV
S65	negative	25.05	positive	nVarIBDV
S66	27.97	negative	positive	non-nVarIBDV
S67	27.44	negative	positive	non-nVarIBDV
S68	31.07	negative	positive	N.D.
S69	24.90	negative	positive	non-nVarIBDV
S70	negative	23.50	positive	nVarIBDV
S71	26.68	negative	positive	non-nVarIBDV
S72	26.96	negative	positive	non-nVarIBDV
S73	25.34	negative	positive	non-nVarIBDV
S74	26.65	negative	positive	non-nVarIBDV
S75	26.49	negative	positive	non-nVarIBDV
S76	29.37	negative	positive	N.D.
S77	25.38	negative	positive	non-nVarIBDV
S78	28.48	negative	positive	N.D.
S79	negative	18.21	positive	nVarIBDV
S80	negative	18.58	positive	nVarIBDV
S81	27.61	negative	positive	non-nVarIBDV
S82	25.41	negative	positive	non-nVarIBDV
S83	26.81	negative	positive	non-nVarIBDV
S84	negative	21.48	positive	nVarIBDV

## Data Availability

All data are reported in this article.
